# Basidiobolomycosis in Togo: clinico-pathological study of a series of 12 presumed cases

**DOI:** 10.1186/s13104-018-3777-8

**Published:** 2018-09-14

**Authors:** Tchin Darré, Bayaki Saka, Abas Mouhari-Toure, Toukilnan Djiwa, Palokinam Pitché, Gado Napo-Koura

**Affiliations:** 1 Department of Pathology, University Teaching Hospital of Lomé, Lomé, Togo; 2 Department of Dermatology, University Teaching Hospital of Lomé, Lomé, Togo; 3 Department of Dermatology, University Teaching Hospital of Kara, Kara, Togo; 4 University of Lomé, BP 1515, Lomé, Togo

**Keywords:** Basidiobolomycosis, *Basidiobolus ranarum*, Histology, Togo, Sub-Saharan Africa

## Abstract

**Objective:**

The purpose of our study was to describe the histological diagnosed of the Basidiobolomycosis cases from 1990 to 2017 (28 years) in the only Pathology Anatomy Laboratory in Togo.

**Results:**

A total of 12 cases of suspected Basidiobolomycosis have been identified. The sex ratio (M/F) was 2. The average age of the patients was 24.8 ± 1.6 years. Six patients (6/12) had a pathological history: HIV infection (n = 4 cases) and tuberculosis (n = 2 cases). The clinical manifestations were localized to pure skin (n = 9 cases), skin and mucous digestive (n = 2 cases) and disseminated (n = 1 cases). Direct mycological examination and culture in 4 patients was positive in 3 patients. The samples examined consisted of 11 cutaneous biopsies measuring 1–3 cm and a biopsy of the intestinal mucosa. Histology showed granulomatous inflammation of the dermohypodermal site with numerous giant cells associated with eosinophilic polynuclear cells, in which there are 5–7 mm non-septate, irregular mycelial filaments. Patients were treated with ketoconazole at a dose of 10 mg/kg daily. The progression of the patients’ condition was favorable after 4 weeks of treatment with a regression of the closets size. Patients were completely healed after 8 weeks of treatment, without recurrence after 6 months. No deaths have been recorded.

## Introduction

Basidiobolomycosis is a rare deep mycosis found in rural areas in tropical areas, mainly in Africa, Asia and Latin America [1]. The main etiologic agents have *Basidiobolus ranarum and Basidiobolus haptosporus*, saprophyte of soil and plants of tropical and subtropical countries [2, 3]. The diagnosis of Basidiobolomycosis is not easy because the clinical and histopathological signs do not point directly to a fungal infection and especially in the tropics where it can simulate a *Mycobacterium ulcerans* infection [4, 5].

Although medical treatment with oral potassium iodide and ketoconazole may be effective, untreated infection may be fatal [5]. In the past 40 years, 179 cases of Basidiobolomycosis have been reported worldwide [4–7]. It is a condition rarely described in Africa and its actual frequency is not known, most published studies being clinical cases [2, 4, 7]. In Togo, there have been 3 published clinical cases, but no serial study is done [2, 5, 8]. This work brings together the cases of Basidiobolomycosis diagnosed in the laboratory of pathological anatomy of Lomé. It aims to clarify the epidemiological, diagnostic and therapeutic aspects of Basidiobolomycosis in Togo.

## Main text

### Methods

This was a descriptive study on all the records (registers and test reports) of histological diagnosed Basidiobolomycosis in the only Laboratory of Pathological Anatomy in Togo, from January 1990 to December 2017. During this period, the samples were recorded in the pathology laboratory register, prepared in fine sections embedded in paraffin (56–60 °C) and then stained with haematin eosin (H.E). The results and review reports of all cases compiled from the registers were collected using a pre-established form. This study was approved by the head of the laboratory department of Sylvanus Olympio teaching Hospital (*Ref N° 08/2017/LAP/CHUSO*). During the counting and data collection patient names were not collected in order to preserve confidentiality.

### Results

Twelve cases of Basidiobolomycosis were diagnosed including 8 men and 4 women. The average age of patients in our series was 24.8 ± 1.6 years, with extremes of 9 and 54 years old. All patients were from rural areas and of low socio-economic status: 9 patients were farmers and 3 students. Six patients (6/12) had a pathological history: HIV infection was known in four patients and two patients ‘was treated for tuberculosis. The clinical manifestations were localized to the skin in 9 cases, coetaneous and digestive mucous in 2 cases and disseminated form in 1 case. In the nine patients with pure skin localization, lesions were located in: lower limbs (n = 6 cases, 4 on the thighs and 2 on the legs), buttocks and legs (n = 2 cases), upper limbs (n = 1 case). The skin lesions were often an infiltrated closet, of firm consistency, with sharp edges, often measuring between 32 cm high and 15 cm wide. These closets were mobilizable compared to the deep planes and not painful to the palpation. There were no satellite lymphadenopathies. The general condition of the patients was preserved. Table 1 summarizes the sociodemographic characteristics of patients.

**Table 1:** Epidemiological characteristics of patients

Characteristics	Values
Sex	
(i) Men	8/12
(ii) Women	4/12
Age (years)	
(i) Average	24.8 ± 1.6
(ii) Extremes	9–54
Profession	
(i) Farmers	9/12
(ii) Students	3/12
Localization	
Skin	9/12
Skin and mucosa	2/12
Disseminated	1/12

The direct mycological examination and culture performed in patients was positive in 3 days between them, with the detection of *B. ranarum*.

From the anatomopathological point of view, the samples examined consisted of 11 cutaneous biopsies measuring 1–3 cm and a biopsy of the intestinal mucosa. Histology had shown tuberculoid granulomas with giant cells, numerous lymphocytes, histiocytes, and eosinophilic cells, an amorphous eosinophilic material also known as the Splendore-Hoeppli phenomenon, and septal hyphae fragments of 10 μm in diameter.

Patients were treated with ketoconazole at a dose of 10 mg/kg daily. Hepatic transaminases were measured at the beginning of treatment and every 2 weeks during treatment. No hepatic intolerance was noted. The progression of the patients’ condition was favorable after 4 weeks of treatment with a regression of the closets size. Patients were completely healed after 8 weeks of treatment, without recurrence after 6 months. No deaths have been recorded.

### Discussion

Our study shows the extreme rarity of Basidiobolomycosis in Togo; however, it is one of the major series reported on this pathology. Microbiological confirmation by PCR for *B. ranarum* and culture provides the most accurate diagnosis, but they are not often available in endemic areas and with varying sensitivity. A combination of histopathological findings, granulomatous inflammation with giant cells, septate hyphal fragments and the Splendore-Hoeppli phenomenon, may confirm Basidiobolomycosis in patients with painless induration of soft tissues [1, 6].

Basidiobolomycosis is endemic in rural intertropical areas, particularly in Indonesia, Burma, India, and sub-Saharan Africa [2, 8]. The infection is caused by the filamentous fungus *B. ranarum*, belonging to the class zygomycetes and entomophthoral order [9]. Zygomycosis is an acute or chronic infection caused by fungal agents belonging to the *phylum Zygomycota* [9]. They are saprophytic fungi and are present in soil, decaying plant material and the intestines of amphibians, reptiles, fish and insectivorous bats [10]. The precise mode of contamination of *B. ranarum* is poorly known, but is thought to be transmitted through the skin after an insect bite, scratch or cut [3, 4].

The diagnosis of Basidiobolomycosis was histological suggested, based on the presence, namely granulomatous inflammation with giant cells, septate hyphal fragments and the Splendore-Hoeppli phenomenon, in patients with painless indurations’ and hard soft tissue [11]. The differential diagnosis of Basidiobolomycosis includes soft tissue tumors, such as synovial sarcoma [11], Hodgkin’s lymphoma [12], and mycetoma [13]. In addition, a tuberculoid granuloma with giant cells can also be observed in Buruli ulcer lesions, especially during healing [7, 8]. However, neither septal hyphal fragments nor the Splendore-Hoeppli phenomenon are observed in Buruli ulcer lesions [8]. The Splendore-Hoeppli phenomenon itself is not specific for Basidiobolomycosis and can also be observed in other infections, such as bronchocentric granulomatosis due to Aspergillus [14], mycetoma [15] and cutaneous Pityrosporum folliculitis [16]. It is the combination of the clinical presentation, the Splendore-Hoeppli phenomenon and the compartmentalized hyphae that suggest Basidiobolomycosis [5, 14]. Cultivation of the *B. ranarum* fungus is difficult and clinical and histopathological features may help to suggest the diagnosis of Basidiobolomycosis [16, 17]. The detection of fungal pathogens by PCR is particularly difficult [17]. Fungal cell walls are not easily lysed for DNA release, leading to false negative PCR results [18]. Isolating *B. ranarum* DNA from formalin-fixed archival tissue blocks and paraffin embedded has been reported, with a protocol allowing reliable purification of fungal DNA [18].

The treatment of Basidiobolomycosis has been based for a long time on potassium iodide, which in most cases gives complete cures in 2–9 months [1–4, 19]. However, the results with this drug are inconsistent. On four patients of the series of Ramesh et al. treated with potassium iodide, only two achieved complete remission after three and 9 months of treatment, respectively [20]. The other two patients, who showed only a simple regression, had to be put on ketoconazole for one, and on itraconazole for the other, to obtain complete remission [19]. In addition to the problem of inconsistent efficacy, this treatment has many side effects [21, 22]. Thus, Madke et al. reported the occurrence of hypothyroidism in a patient during the treatment of Basidiobolomycosis [23].

Azole derivatives have emerged as the treatment of choice for phycomycoses. Among them, ketoconazole has been shown to be effective in Basidiobolomycosis [5, 6, 18]. This imidazole has been used successfully for doses ranging from 5 to 10 mg/kg/day [23, 24]. The duration of treatment for achieving complete cure ranges from 2 to 8 months [24, 25]. No case of therapeutic failure has been reported so far with this antifungal, which has been well tolerated in all reported cases. In addition, none of the publications reported recurrence of symptoms, with periods of decline of 4 months and 11 years [2–7, 25].

## Limitations

The mycological culture to identify the fungus was not done because of technical reasons.

## Abbreviations

H.E: hematoxylin and eoisn
HIV: human immunodeficiency virus
PCR: polymerase chain reaction
DNA: deoxyribonucleic acid
